# Characteristic disease defects in circulating endothelial cells isolated from patients with pulmonary arterial hypertension

**DOI:** 10.1371/journal.pone.0312535

**Published:** 2024-10-28

**Authors:** Kulwant S. Aulak, Lori Mavarakis, Liping Tian, Deborah Paul, Suzy A. Comhair, Raed A. Dweik, Adriano R. Tonelli

**Affiliations:** 1 Department of Immunology and Immunity, Cleveland Clinic, Lerner Research Institute, Cleveland, Ohio, United States of America; 2 Department of Pulmonary, Cleveland Clinic, Allergy and Critical Care Medicine, Respiratory Institute, Cleveland, Ohio, United States of America; Vanderbilt University Medical Center, UNITED STATES OF AMERICA

## Abstract

Pulmonary arterial hypertension (PAH) is a progressive disease characterized by elevated pulmonary arterial pressures that can lead to right heart failure and death. No cure exists for this disease, but therapeutic advancements have extended its median survival from 2 to 7 years. Mechanistic research in PAH has been limited by factors including that a) animal models do not fully recapitulate the disease or provide insights into its pathogenesis, and b) cellular material from PAH patients is primarily obtained from donor lungs during autopsy or transplantation, which reflect end-stage disease. Therefore, there is a need to identify tools that can elucidate the specific mechanisms of human disease in individual patients, a critical step to guide treatment decisions based on specific pathway abnormalities. Here we demonstrate a simple method to isolate and culture circulating endothelial cells (CECs) obtained at the time of right heart catheterization in PAH patients. We tested these CECs using transcriptomics and found that they have typical traits of PAH, including those involving key treatment pathways, i.e. nitric oxide, endothelin, prostacyclin and BMP/activin pathways. CECs show important gene expression changes in other central PAH disease pathways. In summary, we present a new cellular model for the ex-vivo mechanistic evaluation of critical PAH pathways that participate in the pathogenesis of the disease and may help personalized therapeutic decisions.

## Introduction

Pulmonary arterial hypertension (PAH) is a condition characterized by progressive remodeling of the pulmonary arteries leading to increased pressure that if left untreated, results in right heart failure and death [1]. Underlying causes of PAH include heritable factors, connective tissue diseases (CTD), drugs and toxins, portal hypertension, congenital heart diseases, and human immunodeficiency virus. There is also a form without clear etiology named idiopathic PAH (iPAH) [2]. The most common etiologies of PAH are iPAH and PAH-associated with CTD [3,4]. The pathobiology of PAH includes endothelial dysfunction, smooth muscle cell hypertrophy and proliferation, inflammation, metabolic abnormalities, and thrombosis [5–7].

The prognosis of the disease depends on risk assessment and prompt initiation of PAH-specific therapies. Despite effective treatments for PAH the median survival is around 7 years [8]. There is a growing assortment of drugs to treat PAH with limited data to select specific agents. Treatment selection for PAH is currently based on severity of the disease and not on patient-specific molecular defects [2]. Except in the presence of comorbidities, current guidelines indicate dual or triple combination therapy for the treatment of PAH [2]. The recent approval of sotatercept, an agent with a novel mechanism of action, adds a different treatment pathway [9] which further extends the treatment approach [10–12]. A clear understanding of the mechanisms of disease and their therapeutic modification has been the goal in precision medicine [13–15]. Although there has been great progress in understanding of the pathobiology of PAH, mechanistic research has been restricted by critical factors. For instance, animal models cannot replicate the complex and heterogeneous pathogenesis of the human disease [16,17]. This is especially true in iPAH where the cause of the disease is unknown.

Based on the animal model limitations, it remains necessary to carry out studies using biological samples from PAH patients. Cellular material from patients with PAH is primarily obtained from lungs recovered at the time of autopsies or transplantation, which by their nature represent late stages of the disease [18,19]. These cells are of limited supply and may not reflect events that initiate or drive the disease given the potential alteration in their biological responses due to genetic instability, environmental stimuli, and treatment effects [20,21]. There have been attempts to obtain endothelial cells from PAH patients at earlier stages of the disease, including the a) recovery from balloons of pulmonary artery catheters or b) transformation from induced pluripotent stem cells (iPSCs) generated from fibroblast isolated from skin biopsies. These processes can be expensive and technically challenging [22–29], and in the case of iPSC‘s they can introduce new mutations and alter gene expression profiles.

Here we demonstrate a simple method to isolate and culture circulating endothelial cells (CECs) from pulmonary arterial blood obtained at the time of right heart catheterization (RHC) in PAH patients. We tested these endothelial cells using transcriptomics and found that they have the characteristic changes observed in PAH patients, including specific abnormalities in PAH treatment pathways, i.e. nitric oxide, endothelin, prostacyclin and BMP/activin pathways. Furthermore, CECs carried other abnormalities that could advance our understanding of the disease process. Our approach yields CECs with good recovery rates supporting the use of this cellular model for the ex-vivo mechanistic evaluation of critical PAH pathways that participate in the pathogenesis of the disease [30].

## Methods

### Patient selection

We included patients with iPAH or hPAH who underwent RHC for evaluation of PAH and signed the informed consent to participate in our Cleveland Clinic IRB approved study (# 06–245). All blood samples were collected between May 2018 and September 2019. All patients had precapillary PAH, defined as mPAP > 20 mmHg, PAWP ≥ 15 mmHg and PVR ≥ 3 Wood units, based on proceedings of the 6^th^ World Symposium on PH [31]. Patients with iPAH had no identifiable cause that could explain the precapillary PH and all patients with hPAH had either a known genetical abnormality or a first-degree family member with PAH without a clear etiology [2,31].

### Circulating endothelial cell isolation

At the time of RHC, we obtained 10–20 mL of blood in BD Vacutainer® EDTA tubes (BD bioscience, Franklin lakes, NJ) from the distal port of the pulmonary artery catheter, located in the right or left main pulmonary artery. An equal volume of 2% fetal bovine serum (FBS) in phosphate buffered saline (PBS) was added to the blood and the mixture was layered onto 15ml of Ficoll-Paque Plus (Cytiva, Wilmington DE). The sample was centrifuged at 19°C for 30 mins at 740g with maximum acceleration but minimum brake. After centrifugation, the top plasma layer was aspirated and discarded. The buffy coat was then transferred to a new tube, where 14 ml of 2% FBS in PBS was added. This sample was then centrifuged at 19°C for 5 mins at 500g. The supernatant was aspirated and discarded. The cell pellet was resuspended with 5ml 2% FBS in PBS and centrifuged at room temperature for 5 mins at 500g. The supernatant was aspirated, and the pellet resuspended in 2ml of endothelial cell media (EGM-2; Lonza). This sample was then placed onto rat tail collagen (BD bioscience, Franklin lakes, NJ) coated dishes. These plates were prepared by adding 2ml 50ug/ml of rat tail collagen in 0.02N acetic acid into 35mm dishes for 2hrs. After which they were washed twice in PBS and then used to plate isolated cells. The media on the cells was replaced after 24 hours and changed every day for 7 days and then every 48hrs thereafter, until clones appeared (14–21 days). At this stage the only cells that grow in the plates are endothelial cells, as validated by endothelial cell marker, CD31. No further sorting was required to isolate the endothelial cells. After clones appeared and grew sufficiently, they were expanded, used, or frozen for storage.

### Circulating endothelial cell validation

CECs phenotype was confirmed by the endothelial cell-specific marker CD31 (1:30 dilution; Dako, Glostrup, Denmark). Fluorescence-activated cell sorting (FACS) for CD31 expression (Becton Dickinson, San Jose, Calif.) was used as previously described [32]. Cells were also tested for 2D tube formation using an angiogenesis assay kit (#ECM625, Millipore) using approximately 80k cells/well.

### RNA isolation from endothelial cells

All endothelial cells were grown on fibronectin coated plates (50ng/ml) using endothelial cell media, (EGM-2, Lonza Walkerville, MD). Selected cells were passage 5 or 6. Control endothelial cells (cPAEC) from non-PAH subjects were purchased from Lonza. Cells from Lonza were derived from pulmonary arteries from donated lungs, after given permission for their use in research applications by informed consent or legal authorization. When cells were confluent, they were harvested using RNA isolation lysis buffer (RTL buffer + 10ul 2-Mercaptoethanol/ml; Qiagen). RNA was isolated using the RNeasy kit (#74104, Qiagen) and quantitated via NanoDrop and Qubit Assay. The quality of the RNA was assessed using RNA Integrity Number using a bioanalyzer assay.

### RNA sequencing and transcriptomic analyses of endothelial cells

Upon passing quality control, Novogene created sequencing libraries using NEBNext Ultra RNA library prep kit for Illumina (NEB, USA). Between 200ng-1ug RNA was used as input material and libraries were created following the manufacturer’s protocols. Briefly, mRNA was purified from total RNA using poly-T oligos attached to magnetic beads. Fragmentation was carried out using divalent cations under elevated temps in NEBNext first strand synthesis reaction buffer. First strand synthesis was carried out using random hexamer oligos primers and M-MuLV reverse transcriptase. Second strand synthesis was performed using DNA polymerase I and RNAse H and overhangs were converted to blunt ends using exonuclease/polymerase activity. After adenylation of the DNA fragments, NEBnext adaptors with hairpin loop structures were ligated to prepare for hybridization. cDNA fragments of 150bp-200bps were purified using AMPure XF system (Beckman Coulter, Beverly, USA). Then USER enzyme was used with the size selected cDNA at 37°C followed by 5 mins at 95°C before PCR. The PCR was performed with Phusion High- Fidelity DNA polymerase, universal PCR primers and index primers. PCR products were then purified using the AMPure XP system and library quantity assessed on the Agilent Bioanalyzer 2100 system. Original image data files from high-throughput sequencing platforms are available in the NCBI Gene Expression Omnibus (GEO) data repository (accession number GSE277919).

### Transcriptomic analyses of endothelial cells

Clustering and sequencing: Clustering of the index-coded samples was performed using a cBot Cluster Generation System on the PE Cluster Kit cBot-HS (Illumina) according to the manufacturers.

Data analysis-quality control: Raw data of FASTQ format were processed through fastp. Clean data were obtained by removing reads with adapter and poly-N sequences and reads with low quality from raw data (S1–S5 Tables). At the same time, Q20, Q30 and GC content of the clean data were calculated (S1 Table). All the downstream analyses were done on the high-quality clean data. Detailed workflow of RNA sequencing is presented in the (S1 File).

Mapping to reference genome: Reference genome and gene model annotation files were downloaded from the genome browser website (NCBI/UCSC/Ensembl). Clean reads of paired end were aligned to the reference genome using the Spliced Transcripts Alignment to a Reference (STAR) software, that uses sequential maximum mappable seed search in an uncompressed suffix array followed by a seed clustering and stitching procedure. Summary of mapping data for fragment is shown in S3 and S4 Tables.

Quantification of transcripts: Feature Counts were used to calculate the read numbers mapped of each gene. Then Reads Per Kilobase (RPKM) of each gene was calculated based on the length of the gene and reads count mapped to that gene. The summary of the gene expression level analysis (FPKM) is shown in S5 and S6 Tables.

Differential expression analysis: Comparisons were made between CECs and a) control pulmonary artery lung endothelial cells (cPAECs), as well as b) pulmonary lung endothelial cells from PAH patients isolated from patient lungs (LECs). Differential expression analysis between groups were performed using the DESeq2 R package, which provides a model based on the negative binomial distribution. The resulting p values were adjusted using the Benjamini and Hochberg’s approach for controlling the False Discovery Rate (FDR). Genes with an adjusted p value < 0.05 by DESeq2 were assigned as differentially expressed.

### Ingenuity Pathway Analysis (IPA)

Core analysis was performed using IPA for DEGs with adjusted p value (padj) of <0.05 and log2 fold change of ≥ 2. The results of the core analysis for the CECs vs cPAECs were used for comparative analysis. Upstream regulators, downstream analysis, causal networks, and GO analysis was done by the QIAGEN IPA software.

## Results

### A) Isolation of CECs

**Fig 1:**
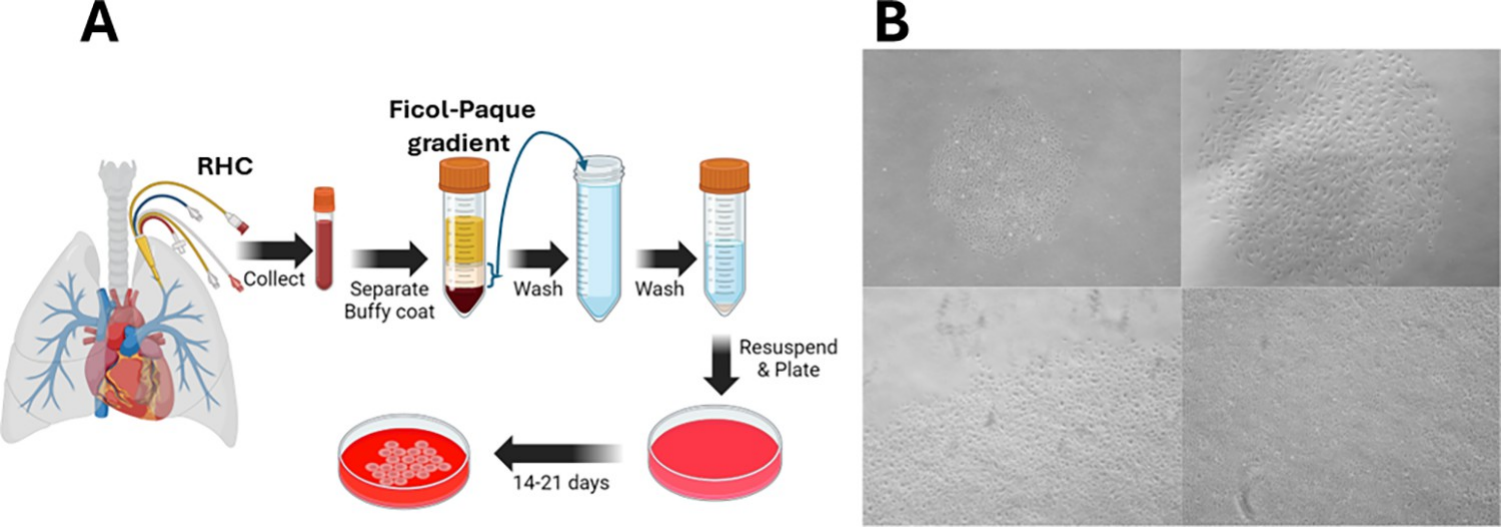
Isolation of CECs. **Panel A** diagrammatic illustration of the CEC isolation procedure. Image was created using BioRender.com. Buffy coats from blood are isolated using a Ficoll-paque gradient and after twice washing cells in FBS/PBS, plated onto collagen coated plates. Clones generally appear after day 14–20. **Panel B** shows representative images of CEC clones.

During RHC, blood was collected from the pulmonary artery and processed as shown in Fig 1A. Representatives images of clones are illustrated in Fig 1B. These cells were CD31+ (Table 1) and produced 2D tubes on Matrigel confirming their endothelial cell behavior. We successfully generated CECs from ~ 60% of patients sampled.

**Table 1:** Patient characteristics.

ID	Type of PH	Age	Gender	Race	% CD31+
**CEC 1**	HPAH	42	F	C	100
**CEC 2**	HPAH	32	F	C	100
**CEC 3**	HPAH	32	F	C	99.8
**CEC 4**	IPAH	74	M	C	100
**CEC 5**	IPAH	42	F	C	100
**CEC 6**	IPAH	34	M	C	99.8
**CEC 7**	IPAH	47	F	C	99.7
**CEC 8**	IPAH	39	M	C	99.5
**LEC 1**	HPAH	56	F	C	99.5
**LEC 2**	IPAH	45	M	C	98.3
**LEC 3**	IPAH	22	F	C	99.0
**LEC 4**	HPAH	41	M	C	99.0
**LEC 5**	HPAH	50	F	C	98.0
**cPAEC 1**	Control	65	M	C	
**cPAEC 2**	Control	21	M	H	
**cPAEC 3**	Control	67	F	C	
**cPAEC 4**	Control	52	F	H	
**cPAEC 5**	Control	44	F	AA	

**Abbreviations:** CEC: Circulating endothelial cells, LEC: PAH Lung derived endothelial cells, cPAECs: Control lung endothelial cells, HPAH: Hereditary pulmonary arterial hypertension, IPAH: Idiopathic pulmonary arterial hypertension.

### B) Transcriptomic analysis of CECs and LECs in PAH patients

**Fig 2:**
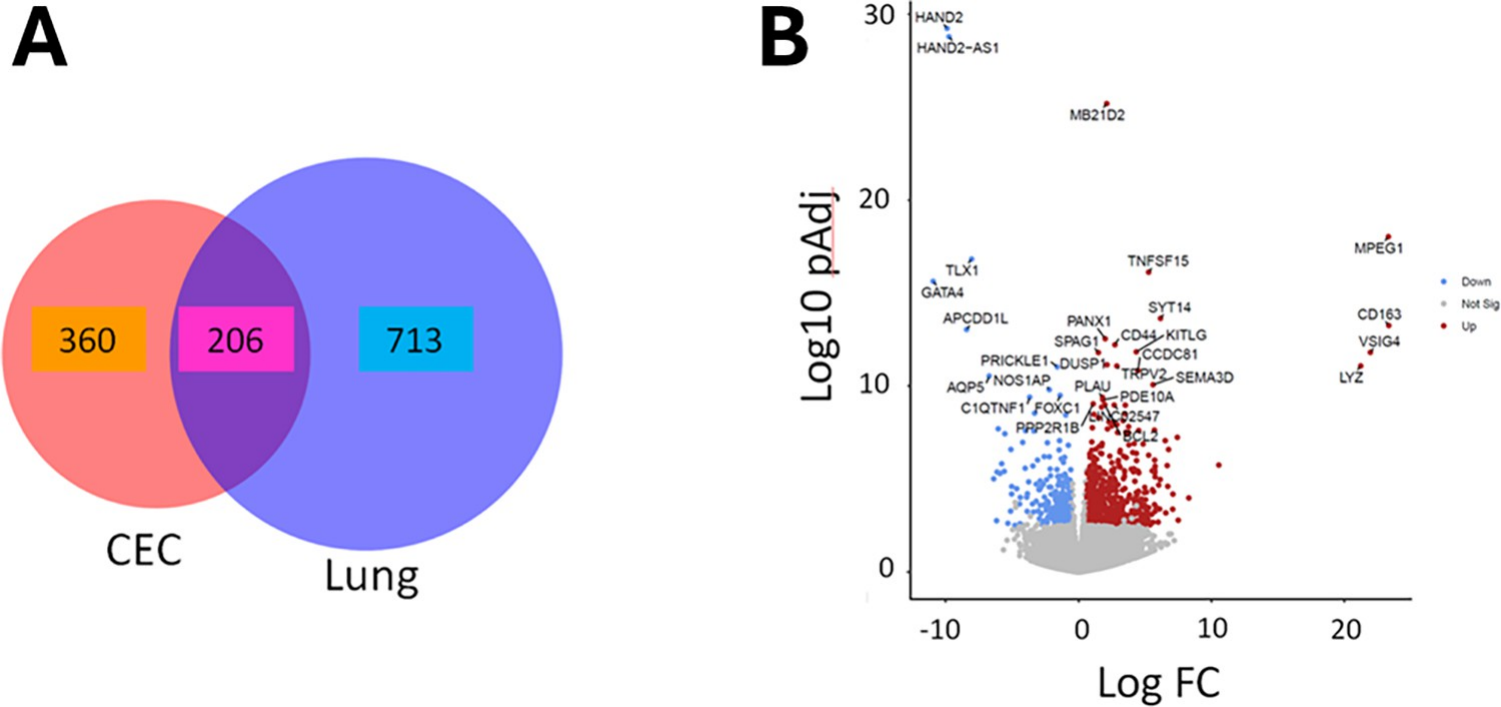
Gene expression in PAH CECs and LECs compared to control PAEC. **Panel A** shows a Venn diagram displaying differentially expressed genes (DEGs) in CECs and LECs compared to cPAECs. We found an overlap in 206 genes between CECs and LECs supporting the similarity between the cell lines. **Panel B** represents a Volcano plot showing the distribution of DEGs between CECs vs cPAECs. The top 30 altered DEGs are labeled.

**Fig 3:**
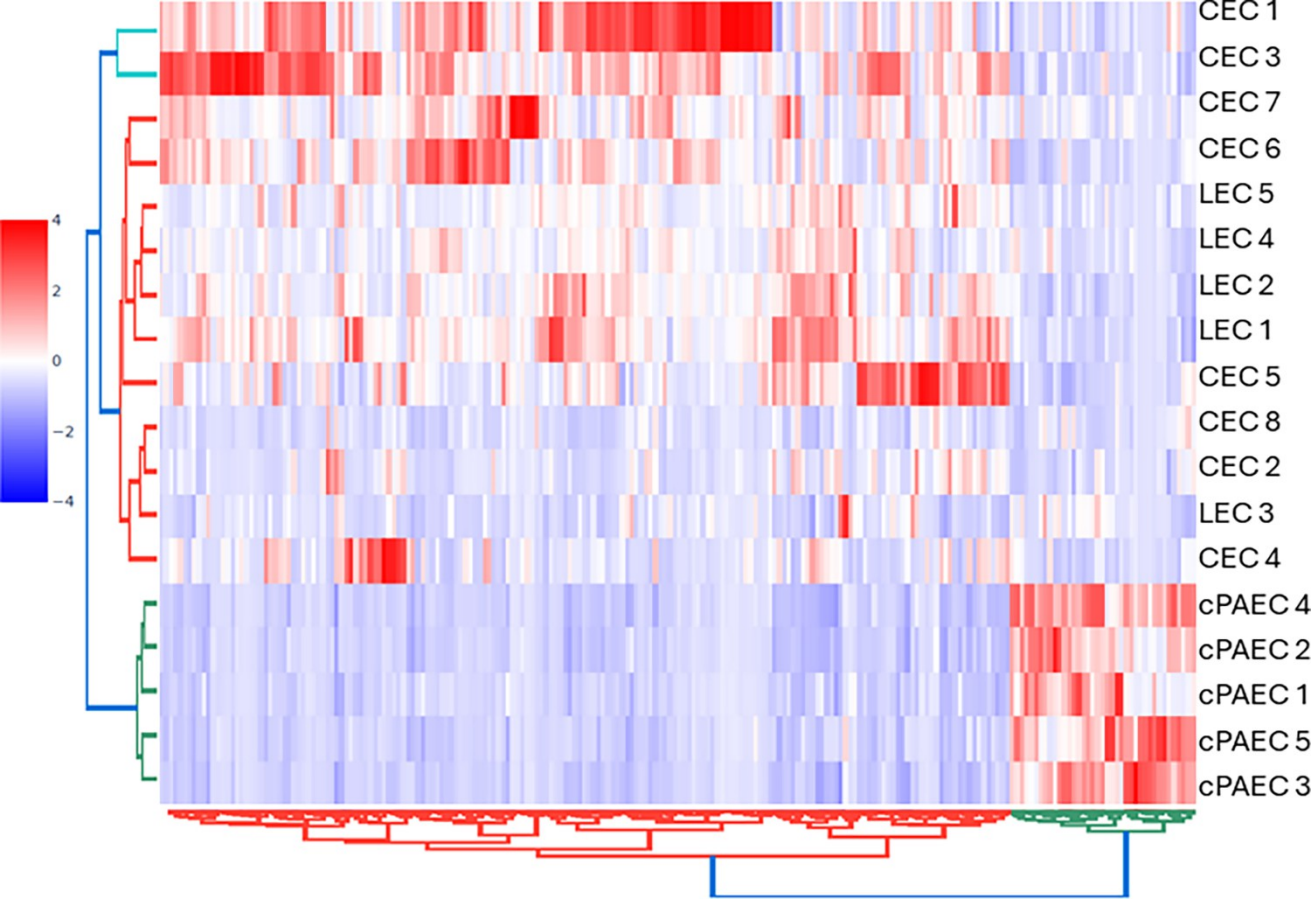
Heat map of genes altered in CECs and LECs. CECs and LECs from PAH patients were compared to cPAECs. Using hierarchical clustering, we show that DEG shows similarities between CECs and LECs, while revealing differences with cPAECs. Genes that were significantly different (pAdj<0.05) and with a Log2FC>2 were used to generate a heat map. Hues of red color reflect DEG upregulation, while shades of blue color reflect downregulation.

We compared eight CECs and five LECs to five cPAEC (Table 1). When compared to cPAECs, we observed 566 differentially expressed genes (DEGs) that were significantly altered two-fold in CECs (padj<0.05), while 919 genes were altered two-fold in LECs and 206 were common in both groups, (Fig 2A). In CECs, 426 genes were upregulated, and 140 genes were downregulated, while, in LECs, 670 genes were upregulated, and 249 genes were downregulated. A volcano plot shows the top 30 genes that are altered in CECs (Fig 2B), with many involved in signaling, proliferation and clotting. A hierarchical clustered heat map of DEGs from CECs and LECs shows difference between cPAEC (Fig 3).

### C) Differential expression of endothelial cells markers on isolated cells

To verify that the isolated cells were of endothelial origin, we checked the endothelial cell markers expression in CECs/LECs vs cPAECs. No statistical difference was observed between CECs or LECs in the pan-endothelial cell markers or arterial endothelial cells markers (Table 2). Due to low expression and variability of some genes, the fold differences may have been high but not significant. Data on the expression of genes shown here are provided in S7 Table. Interestingly, vasoactive intestinal peptide receptor 1 (VIPR1) (Table 2), a marker for lung specific endothelial cells know to be downregulated in PAH patients [33], was greatly reduced in CECs (log2FC = -4.21, pAdj = 3.09E-05) and in LECs (log2FC = -0.7582 pAdj = 0.21).

**Table 2:** Differential gene expression of endothelial markers in CECs or LEC compared to cPAEC.

		CEC		LEC	
Gene	log2FC	padj	log2FC2	padj3	
PECAM1	0.187	0.662	-0.164	0.685	
CHD5	0.497	0.88	0.272	0.85	** **
VWF	-0.501	0.708	0.789	0.202	**PAN**
KDR	-0.214	0.668	-0.047	0.938	
FLT1	0.764	0.128	-0.139	0.663	** **
TEK	0.361	0.312	-0.157	0.351	** **
CLDN5	-0.158	0.923	0.337	0.723	** **
IL7R	4.076	1.00E-04	2.380	0.001	**Lung endothelial cells**
EDNRB	-1.596	0.211	-0.105	0.956	
GPIHBP1	-0.883	0.596	-0.154	0.946	
CD36	1.399	0.657	-0.262	0.920	
S100A4	-0.459	0.808	-0.197	0.850	
PTPRB	-0.170	0.822	-0.455	0.242	
EDN1	-0.271	0.823	0.292	0.556	
FCN3	-0.272	0.935	-3.942	1.000	
APLN	0.113	0.943	0.196	0.774	
SOSTDC1	1.794	1.000	2.390	1.000	** **
SOX17	0.3492	0.6726	0.8263	5.63E-06	
GJA5	1.1076	0.6399	3.3781	0.0006	**Artery Specific**
GATA2	-0.5493	0.319	0.9574	0.0026	
CXCR4	-0.7453	0.4593	0.7115	0.25	
MECOM	0.3722	0.2877	-0.1336	0.6212	
HEY1	0.9543	0.1826	-0.1986	0.7798	
GJA4	-2.2373	0.2561	-0.1966	0.9195	
SERPINE2	1.9644	0.0306	0.1648	0.8311	
DKK2	-2.9263	0.2527	0.829	0.7349	

### D) Differential gene expression between circulating endothelial cells and PAH Lung endothelial cells

The top 20 genes that are up- and down-regulated in CECs and LECs are shown in **Table 3**. The most down-regulated genes in both CECs and LECs were the transcription factors GATA4 (CECs- Log2FC = -10.94, pAdj = 5.77E-13; LECs- Log2FC = -11.02, pAdj = 3.09E-11) and heart and neural crest derivatives expressed 2 (HAND2: CECs- Log2FC = -9.90, pAdj = 1.01E-25; LECs- Log2FC = -9.98, pAdj = 6.06E-19). These two proteins are involved in embryonic development and adult cell homeostasis. Defects in HAND2 can cause pathological cardiac remodeling [34–36]. HAND2 also interacts with NK2 transcription factor related locus 5 (NKX2-5) [37], which was also decreased in CECs (Log2FC = -5.51, pAdj = 1.18E-05 and LECs (Log2FC = -6.90, pAdj = 1.44E-08). Both GATA4 and NKX2-5 were also found to be reduced in endothelial cells isolated from pulmonary artery catheters balloons of PAH patients [23].

**Table 3:** Top 20 DEG in CECs and LEC compared to cPAECs.

CEC							LEC						
Gene	log2FC	padj		Gene	log2FC	padj	Gene	log2FC	padj		Gene	log2FC	padj
GATA4	-10.9382	5.77E-13		CD163	23.3318	1.07E-10	GATA4	-11.0155	3.09E-11		ADGRG7	25.0229	1.36E-13
HAND2	-9.9012	1.01E-25		MPEG1	23.3025	4.02E-15	HAND2	-9.9793	6.06E-19		GLI2	8.1700	3.07E-02
APCDD1L	-8.4261	1.57E-10		VSIG4	21.9501	1.81E-09	MRC1	-8.9690	2.88E-08		SLC22A31	7.9423	2.46E-06
TLX1	-8.0480	5.16E-14		LYZ	21.2194	8.07E-09	TLX1	-8.1263	2.56E-11		CDH6	7.7810	1.55E-09
AQP5	-6.7229	2.41E-08		GLI2	10.5459	2.97E-04	HRH2	-7.6590	1.76E-07		VWDE	7.3293	3.92E-10
IRX3	-6.3861	1.02E-03		EPHA5	8.2910	5.46E-03	CSMD1	-7.2969	2.00E-03		SLC6A15	7.1442	9.70E-11
SALL3	-6.1719	3.50E-02		ATP6V0D2	7.4940	3.36E-02	IRX3	-7.1792	9.60E-03		PRSS12	6.5840	1.34E-04
TFF3	-6.1375	5.25E-04		SLC6A15	7.4304	1.77E-05	IRX5	-6.9825	7.12E-07		PDE1C	6.4840	1.33E-03
APCDD1	-6.0466	7.62E-06		PRSS12	7.0962	3.89E-03	TFF3	-6.9392	3.99E-03		TNC	6.3866	9.39E-05
SLC16A12	-5.9125	5.99E-04		UBE2QL1	7.0766	1.44E-02	NKX2-5	-6.8924	1.44E-08		ZNF804A	6.1822	3.10E-05
HRH2	-5.7838	2.55E-04		FMN1	6.7686	6.34E-05	APLNR	-6.7514	8.27E-03		FRMPD4	5.9229	2.12E-03
NKX2-5	-5.5480	1.18E-05		SLC16A9	6.7059	2.02E-03	GRIA4	-6.3497	4.30E-06		DIO2	5.6922	1.22E-06
CALHM4	-5.3158	4.24E-02		BCHE	6.6633	3.07E-04	LRMP	-5.3132	3.55E-03		GBP5	5.6798	5.44E-06
GALNT8	-5.1132	1.69E-02		CD69	6.5608	1.94E-02	IGFBP2	-4.9068	1.22E-04		AC008687.4	5.6454	1.99E-04
GRIA4	-5.0934	6.34E-05		GADL1	6.5139	2.59E-05	POF1B	-4.8295	6.91E-05		TPO	5.5242	1.99E-04
SLIT3	-5.0373	3.85E-03		SYT14	6.1593	4.97E-11	GYPE	-4.8105	1.03E-05		CNTN1	5.4260	1.22E-05
IRX5	-5.0304	2.03E-03		CDH6	6.1348	1.08E-03	NUP210	-4.7289	2.96E-05		NPPB	5.3380	4.37E-03
NPTX2	-4.7910	4.91E-02		NPTX1	6.0931	1.42E-02	MLPH	-4.2839	5.11E-03		CLDN1	5.2887	4.12E-03
CPXM1	-4.6601	2.46E-03		FGF1	6.0247	1.75E-03	FGR	-3.7832	1.62E-02		AFF3	5.2820	2.30E-05
VIPR1	-4.2054	3.09E-05		KCNQ3	5.9852	3.91E-02	ZNF730	-3.7689	9.75E-04		PAPPA	5.2346	5.76E-03

Green shaded genes are down regulated and blue shaded genes are upregulated in CECs and LECs compared to cPAECs.

### E) Gene Ontology analysis on differentially expressed genes in circulating endothelial cells

Functional pathway enrichment for DEGs with greater than 2-fold change was carried out in CECs and LECs using Gene Ontology (GO) analysis. Results from the top biological and cellular processes are presented in Table 4. For both CECs and LECs, biological processes enrichment showed that the processes predominantly affected were angiogenesis, cell growth, blood circulation, and heart development, which are important in the pathobiology of PAH. In the molecular function analysis, the receptor regulatory activity involved in PAH was enriched in both CECs and PAH PAECs.

**Table 4:** Functional enrichment of DEG in CECs and LECs.

	CEC’s GO analysis				LECs GO analysis		
GO -BPs	Description	Count	padj	GO -BPs	Description	Count	padj
GO:0051272	positive regulation of cellular component movement	43	2.76E-05	GO:0001525	angiogenesis	62	8.74E-07
GO:0003013	circulatory system process	42	4.82E-06	GO:0048598	embryonic morphogenesis	60	3.80E-05
GO:0030335	positive regulation of cell migration	42	2.24E-05	GO:0051272	positive regulation of cellular component movement	54	3.20E-04
GO:0008015	blood circulation	41	4.82E-06	GO:0030855	epithelial cell differentiation	54	8.50E-04
GO:0044057	regulation of system process	41	6.62E-06	GO:0007409	axonogenesis	50	1.60E-04
GO:0007507	heart development	39	3.60E-04	GO:0048568	embryonic organ development	48	4.10E-05
GO:0050865	regulation of cell activation	36	9.00E-04	GO:0002521	leukocyte differentiation	48	4.20E-04
GO:0001525	angiogenesis	35	2.08E-03	GO:0010720	positive regulation of cell development	48	6.17E-03
GO:0016049	cell growth	34	2.36E-03	GO:0007507	heart development	47	1.38E-02
GO:0070371	ERK1 and ERK2 cascade	24	3.02E-03	GO:0022604	regulation of cell morphogenesis	46	5.46E-03
				GO:0016049	cell growth	45	7.72E-03
**GO-CCs**	**Description**	**Count**	**padj**				
GO:0098797	plasma membrane protein complex	37	5.70E-04	**GO -CCs**	**Description**	**Count**	**padj**
GO:0005911	cell-cell junction	32	1.12E-03	GO:0031012	extracellular matrix	42	1.60E-05
GO:0045121	membrane raft	28	3.50E-04	GO:0005788	endoplasmic reticulum lumen	33	3.47E-03
GO:0098589	membrane region	28	3.70E-04	GO:0044420	extracellular matrix component	18	8.98E-03
GO:0043235	receptor complex	25	9.72E-03	GO:0005578	proteinaceous extracellular matrix	17	1.11E-02
GO:0045177	apical part of cell	23	3.45E-02	GO:0005604	basement membrane	15	1.11E-02
GO:0031253	cell projection membrane	21	4.22E-02	GO:0005871	kinesin complex	11	1.94E-02
GO:1902495	transmembrane transporter complex	20	1.30E-02	GO:0000940	condensed chromosome outer kinetochore	5	2.96E-02
GO:0030667	secretory granule membrane	19	4.37E-02				
GO:0034702	ion channel complex	19	1.30E-02				
**GO-MFs**	**Description**	**Count**	**padj**	**GO -MFs**	**Description**	**Count**	**padj**
GO:0022890	inorganic cation transmembrane transporter activity	31	4.72E-02	GO:0000978	RNA polymerase II proximal promoter DNA binding	44	1.77E-02
GO:0030545	receptor regulator activity	29	5.10E-03	GO:0000982	DNA-binding TF activity, RNA polymerase II-specific	43	1.37E-03
GO:0048018	receptor ligand activity	27	5.10E-03	GO:0030545	receptor regulator activity	42	3.20E-04
GO:0046873	metal ion transmembrane transporter activity	27	2.04E-02	GO:0001228	transcriptional activator activity	39	1.31E-02
GO:0005539	glycosaminoglycan binding	16	4.72E-02	GO:0005201	extracellular matrix structural constituent	24	2.20E-04
GO:0008083	growth factor activity	14	2.56E-02	GO:0005539	glycosaminoglycan binding	23	1.83E-02
GO:0005244	voltage-gated ion channel activity	14	4.72E-02	GO:0005126	cytokine receptor binding	23	4.24E-02
GO:0022832	voltage-gated channel activity	14	4.72E-02	GO:0019838	growth factor binding	17	4.65E-02
GO:0005201	extracellular matrix structural constituent	13	4.72E-02	GO:0019955	cytokine binding	15	1.86E-02

**Abbreviations:** GO, Gene Ontology; BP, Biological Process; CC, Cellular Components; MF, Molecular Functions.

### F) Upstream regulator analysis

We analyzed upstream regulators which can identify transcriptional factors affecting the DEG. In CECs, the top upstream regulators of DEG were tumor necrosis factor (TNF; Z score = 7.84), interferon ƴ (IFNG; Z score = 6.26), thrombin (F2; Z score = 5.79) and transforming growth factor β1 (TGFB1; Z score = 5.44). Proteins known to play a significant role in PAH such as epidermal growth factor, SMAD3 (Z score = 4.48), endothelin 1 (EDN1- Z score = 4.34), hepatocyte growth factor (Z score = 4.19), and vascular endothelial growth factor A (VEGFA; Z score = 4.11) (Table 5) were activated [38–42].

**Table 5:** Ingenuity pathway analysis of highly affected networks in CECs.

Upstream Regulator	Expr Log Ratio	Molecule Type	Predicted Activation State	Activation z-score	p-value of overlap	Upstream Regulator	Expr Log Ratio	Molecule Type	Predicted Activation State	Activation z-score	p-value of overlap
TNF	1.227	cytokine	Activated	7.484	2.32E-17	RNASEH2B	0.049	other	Inhibited	-5.056	5.17E-10
IFNG		cytokine	Activated	6.26	1.16E-19	ETV3	0.361	transcription regulator	Inhibited	-4.583	7.54E-12
F2	0.738	peptidase	Activated	5.788	5.16E-10	ETV6	-0.365	transcription regulator	Inhibited	-4.437	6.39E-14
TGFB1	0.262	growth factor	Activated	5.436	4.13E-14	LACTB	-0.305	peptidase	Inhibited	-3.961	1.73E-12
RRAS2	0.995	enzyme	Activated	5.196	8.45E-09	Ttc39aos1		other	Inhibited	-3.717	9.12E-07
IRF7	1.333	transcription regulator	Activated	5.181	1.29E-09	IMMT	-0.026	other	Inhibited	-3.606	1.16E-07
IL1B	-0.51	cytokine	Activated	5.089	1.15E-08	CITED2	0.547	transcription regulator	Inhibited	-3.515	1.40E-06
STAT1	0.117	transcription regulator	Activated	4.971	2.40E-10	MYCN	-2.346	transcription regulator	Inhibited	-3.508	2.53E-05
RIPK2	0.787	kinase	Activated	4.909	2.58E-10	STAG2	-0.216	other	Inhibited	-3.477	9.43E-09
IFNA2		cytokine	Activated	4.9	1.07E-11	NKX2-3	-4.444	transcription regulator	Inhibited	-3.28	1.92E-11
NFkB		complex	Activated	4.834	1.00E-06	SIRT1	0.317	transcription regulator	Inhibited	-3.268	8.48E-04
IL5		cytokine	Activated	4.707	1.26E-04	SP110	0.529	transcription regulator	Inhibited	-3.207	2.06E-02
NONO	-0.427	transcription regulator	Activated	4.61	1.73E-11	BANF1	-0.068	other	Inhibited	-3.2	5.15E-05
IL1A	1.524	cytokine	Activated	4.566	1.13E-06	UBAP1	-0.124	other	Inhibited	-3.148	1.31E-06
MYD88	-0.039	other	Activated	4.521	4.87E-05	USP18	1.275	peptidase	Inhibited	-3.132	5.71E-04
IRF1	-0.11	transcription regulator	Activated	4.515	8.19E-10	PTPN6	1.511	phosphatase	Inhibited	-3.063	1.27E-03
EGF	2.254	growth factor	Activated	4.489	1.79E-05	TRIM24	0.88	transcription regulator	Inhibited	-3.053	2.24E-04
SMAD3	0.148	transcription regulator	Activated	4.48	5.44E-09	SOCS1	-0.104	other	Inhibited	-2.962	6.16E-07
IFNAR1	0.349	TM receptor	Activated	4.477	2.72E-07	RUNX3	4.003	transcription regulator	Inhibited	-2.866	9.52E-05
RELA	0.456	transcription regulator	Activated	4.464	1.58E-04	DACH1	0.071	transcription regulator	Inhibited	-2.789	7.47E-04
CSF2	1.661	cytokine	Activated	4.345	1.72E-05	F3	1.884	transmembrane receptor	Inhibited	-2.716	3.12E-07
EDN1	-0.271	cytokine	Activated	4.335	1.95E-08	ETV7	2.156	transcription regulator	Inhibited	-2.646	2.50E-03
IL27	0.738	cytokine	Activated	4.307	2.00E-07	MEOX2	-0.199	transcription regulator	Inhibited	-2.644	3.28E-06
IFN Beta		group	Activated	4.3	1.34E-07	BMAL1	0.211	transcription regulator	Inhibited	-2.63	7.79E-02
IFNL1		cytokine	Activated	4.267	2.72E-10	ZNF217	0.483	transcription regulator	Inhibited	-2.63	7.98E-03
TICAM1	0.266	other	Activated	4.199	1.08E-05	NCOR1	-0.05	transcription regulator	Inhibited	-2.621	1.46E-03
HGF	0.031	growth factor	Activated	4.19	1.18E-08	FOXA1	2.324	transcription regulator	Inhibited	-2.608	3.41E-06
TLR3	0.22	transmembrane receptor	Activated	4.136	8.26E-06	SATB1	-0.634	transcription regulator	Inhibited	-2.519	5.19E-04
XBP1	0.483	transcription regulator	Activated	4.122	3.39E-02	ZFP36	-0.218	transcription regulator	Inhibited	-2.495	3.35E-02
VEGFA	0.686	growth factor	Activated	4.107	6.04E-06	APOE	-0.168	transporter	Inhibited	-2.481	4.88E-03

### G) Causal network analysis

Causal network analysis demonstrated that the top master regulator was activin A with a depth of 3 (Z score = 7.63; pval of overlap = 1.64E-36). Activin A is elevated in PAH and downregulates BMPR2 by causing its degradation (**Table 6**) [43,44]. We also observed an increase in activin A (INHBA) transcription in CECs (log2FC = 2.53, pAdj = 0.04) and LECs (log2FC = 2.09, pAdj = 0.09).

**Table 6:** Ingenuity pathway analysis of causal networks.

Master Regulator	Expr Log Ratio	Molecule Type	Depth	Predicted Activation	Activation z-score	p-value of overlap	Master Regulator	Expr Log Ratio	Molecule Type	Depth	Predicted Activation	Activation z-score	p-value of overlap
TLR5	0.715	transmembrane receptor	2	Activated	10.29	1.11E-30	RPL22	-0.386	translat. regulator	3	Inhibited	-9.033	2.41E-39
TAT	0.738	enzyme	2	Activated	9.979	8.82E-45	UBE3C	0.104	enzyme	3	Inhibited	-9.014	1.94E-27
TNF	1.227	cytokine	1	Activated	9.397	3.44E-42	PDCD1		TM receptor	2	Inhibited	-8.875	2.39E-27
TRPC4AP	0.204	transporter	2	Activated	9.381	4.01E-23	LPCAT1	-0.091	enzyme	3	Inhibited	-8.852	1.68E-39
IRF3/IRF7		complex	3	Activated	8.972	6.17E-28	mir-302	-1.938	microRNA	3	Inhibited	-8.733	1.33E-26
RETREG1	0.364	other	3	Activated	8.967	6.30E-39	TRIM21	0.717	enzyme	2	Inhibited	-8.171	6.35E-28
PARP16	-0.458	enzyme	3	Activated	8.904	7.16E-38	FBXL20	-0.229	other	3	Inhibited	-7.923	1.59E-22
CLEC7A	4.759	transmembrane receptor	2	Activated	8.903	1.09E-26	SPOP	-0.304	other	2	Inhibited	-7.632	3.55E-27
MAILR	0.423	other	3	Activated	8.891	1.02E-26	ANXA5	0.042	transporter	2	Inhibited	-7.492	2.05E-25
IRF3	0.145	transcription regulator	2	Activated	8.835	4.91E-26	MSR1	2.742	TM receptor	2	Inhibited	-7.484	2.86E-22
IFNE	1.964	cytokine	2	Activated	8.733	1.76E-27	PHLPP1	0.639	enzyme	2	Inhibited	-7.39	5.16E-28
TNF	1.227	cytokine	2	Activated	8.588	4.21E-48	USP46	0.341	peptidase	3	Inhibited	-7.309	3.36E-28
CCDC85A	0.729	other	3	Activated	8.376	3.05E-35	SRP54	0.239	enzyme	3	Inhibited	-7.067	3.63E-33
F3/F7		complex	2	Activated	8.353	1.76E-24	USP19	-0.277	peptidase	2	Inhibited	-7.02	3.81E-22
ERN1	-0.242	kinase	2	Activated	8.256	5.01E-33	ANXA5	0.042	transporter	3	Inhibited	-6.828	3.82E-32
IFNL1		cytokine	2	Activated	8.165	1.34E-26	miR-16-5p		mature microRNA	2	Inhibited	-6.6	9.09E-23
CTH	-0.101	enzyme	2	Activated	8.109	5.32E-28	SLC39A10	0.34	transporter	2	Inhibited	-6.593	1.99E-21
F2	0.738	peptidase	1	Activated	8.021	2.62E-23	mir-137		microRNA	2	Inhibited	-6.423	3.40E-19
**Activin A**		**complex**	**3**	**Activated**	**7.625**	**1.64E-36**	BAP1	-0.236	peptidase	2	Inhibited	-6.377	1.14E-25
TNK2	-0.454	kinase	2	Activated	7.493	3.11E-21	miR-126a-5p		mature microRNA	2	Inhibited	-6.325	4.41E-20
BMAL1:CLOCK,		complex	3	Activated	7.441	5.83E-29	GATA5	-2.255	transcript regulator	2	Inhibited	-6.273	4.37E-17
CD38	-0.258	enzyme	2	Activated	7.357	7.87E-30	BHLHE41	1.668	transcript. regulator	3	Inhibited	-6.251	2.00E-17
CRAC channel		complex	3	Activated	7.333	5.20E-36	LRBA	-0.376	other	2	Inhibited	-6.231	4.95E-20
HTR1B	-1.298	GPCR	2	Activated	7.298	5.74E-32	RNF5	-0.074	enzyme	3	Inhibited	-6.205	3.98E-30
IRF7	1.333	transcription regulator	2	Activated	7.249	6.73E-13	miR-615-3p (CCGAGCC)		mature microRNA	2	Inhibited	-6.194	3.13E-18
KSR1	-0.447	kinase	2	Activated	7.247	3.42E-27	CLIC2	0.01	enzyme	3	Inhibited	-6.188	8.66E-21
AFAP1-AS1	0.147	other	3	Activated	7.233	5.45E-13	ZDHHC6	0.152	enzyme	2	Inhibited	-6.174	5.49E-19
Nodal receptor		complex	3	Activated	7.2	1.35E-40	MARCHF5	0.58	enzyme	2	Inhibited	-6.173	1.06E-10
PROM1	-0.987	other	2	Activated	7.115	2.79E-21	ANGPTL1	0.188	other	2	Inhibited	-6.129	5.68E-19
IKBKE	0.8	kinase	2	Activated	7.114	1.09E-33							
COL4A4	0.034	other	3	Activated	7.102	2.53E-32							
PDK		group	2	Activated	7.09	1.80E-31							

### H) Alterations of genes important in PAH treatment pathways

Several vasodilator treatments are available in PAH that target nitric oxide, prostacyclin, endothelin and activin pathways. We investigated if defects in these pathways are present in CECs or LECs.

**1) Nitric oxide pathway**: In both CECs and LECs, we found no significant difference in gene expression of the endothelial form of nitric oxide synthase (eNOS) (NOS3); however, cellular levels of eNOS are regulated by other factors such as CAV1 which stabilizes eNOS and correctly localize it into caveolae [45,46]. We found lower levels of cav-1 in CECs (Log2FC = -0.57, pAdj = 0.07) and LECs (log2FC = -0.86, pAdj = 1.49E-8), that would lead to decreased stability of eNOS, and/or decreased phosphorylation needed for eNOS activity [46–49].

Critical downstream components of NO signaling pathway include phosphodiesterase’s (PDEs). We found higher levels of PDE5 in both CECs (log2FC = 1.68, pAdj = 0.003) and LECs (log2 FC = 1.09, pAdj = 0.02) which are responsible for down regulating NO signaling [50,51]. In addition, we saw differences in other PDE enzymes that play a role in this and other signaling pathways [50,52,53], including, increased levels of PDE10A in CECs (log2FC = 1. 1.83, pAdj = 3.601E-07) and LECs (log2 FC = 0.85, pAdj = 0.009), and decreased levels of PDE3A in CECs (log2FC = -1.34, pAdj = 0.06) and LECs (log2FC = -1.25, pAdj = 0.0003) and PDE7B in CECs (log2FC = -1.28, pAdj = 0.04) and LECs (log2FC = -0.36, pAdj = 0.31) (**Table 7**).

**Table 7:** Differential expression of genes involved in PAH from CEC or LEC compared to cPAEC.

	CEC		LEC			
Gene	log2FC	padj	log2FC	padj	Pathways affected	Molecule Type
NOS3	-0.2214	0.8461	-0.1858	0.6965	Nitric Oxide	Enzyme
CAV1	-0.564	0.066	-0.8642	1.49E-08	Nitric Oxide	scaffolding protein
PDE5A	1.6781	0.0028	1.0945	0.0196	Nitric Oxide	Phosphodiesterase
PTGIR	-1.4576	0.0158	-0.2034	0.7486	Prostaglandin	Receptor
PTGER4	1.4283	0.0181	1.2954	0.0052	Prostaglandin	Receptor
PTGER1	1.98	0.4137	3.5182	0.0014	Prostaglandin	Receptor
GNAI1	1.9431	0.0316	0.745	0.2558	Prostaglandin	G protein
PTGS2	3.631	0.0076	0.3851	0.5288	Prostaglandin	Enzyme
PTGR1	-0.8936	0.0392	-1.043	0.0003	Prostaglandin	Enzyme
TBXA2R	2.3605	0.0143	1.6069	0.0033	Prostaglandin	Receptor
PDE7B	-1.283	0.0397	-0.3578	0.3095	Prostaglandin	Phosphodiesterase
SMURF1	0.5236	0.0747	0.5911	0.0009	BMP/Activin	Inhibitor
SMURF2	1.5732	0.0004	1.0124	0.0156	BMP/Activin	Inhibitor
INHBA	2.531	0.0392	2.09	0.0857	BMP/Activin	Inhibitor
BMPR2	0.75	0.1373	-0.6958	0.0333	BMP/Activin	Receptor
ACVRL1	0.7816	0.0049	0.7582	5.40E-07	BMP/Activin	Receptor
TGFBR1	1.5006	0.0069	0.2503	0.3232	BMP/Activin	Receptor
ACVR1	0.8163	0.0381	0.151	0.4255	BMP/Activin	Receptor
NOG	4.3712	0.0195	2.4346	0.0412	BMP/Activin	Inhibitor
TWSG1	0.9057	0.031	0.272	0.1164	BMP/Activin	inhibitor
SMAD6	1.898	0.052	0.869	0.0087	BMP/Activin	Inhibitor
PDE10A	1. 1.825	3.60E-07	0.8542	0.0091	Nitric Oxide/prostaglandin	Phosphodiesterase
PDE3B	-2.3542	0.0073	-0.339	0.5282	Nitric Oxide/prostaglandin	Phosphodiesterase
PDE3A	-1.3421	0.0618	-1.2464	0.0003	Nitric Oxide/prostaglandin	Phosphodiesterase
VIPR1	-4.2054	3.09E-05	-0.7582	0.2108	Inflammation/vasorelaxation	Receptor
NEDD9	2.31	0.0032	1.55	5.75E-06	Apoptosis	scaffolding protein
DKK1	1.7705	3.42E-05	0.976	0.0001	Wnt	Inhibitor
DKK3	2.6697	0.0004	1.043	0.062	Wnt	Inhibitor
CXCL1	1.7693	0.0075	1.983	3.59E-05	Inflammation	Ligand
IL-8	3.745	0.0057	2.3956	0.0183	Inflammation	Ligand
IL7R	4.0759	8.59E-05	2.3798	5.08E-04	Inflammation	Ligand
ENTPD1	-1.4123	0.0246	-1.009	0.0195	Purinergic	Ligand

**2) Prostacyclin pathway:** We found that CECs have reduced gene expression of the prostacyclin receptor (PTGIR) (log2FC = -1.46, pAdj = 0.02), consistent with the reduced levels observed in PAH patients [54]. Other prostaglandin receptors are also altered in PAH [55] and we noted that in both CECs and LECs there was increased expression of the PGE_2_ receptor, EP4 (PTGER4) in CECs (log2FC = 1.43, pAdj = 0.02) and LECs (log2 FC = 1.30, pAdj = 0.005). EP1 (PTGER1), another PGE_2_ receptor, was elevated over 11-fold in LECs (Log2FC = 3.52, pAdj = 0.001) while it was unchanged in CECs, possibly suggesting patient or disease stage differences. We saw an increased levels of the thromboxane A2 receptor (TBXA2R) in CECs (log2FC = 2.36, pAdj = 0.014) and LECs (log2 FC = 1.601, pAdj = 0.003). Thromboxane A2 is prostanoid that causes vasoconstriction and is elevated in PAH [56]. In CECs we saw an increase in Cox-2 expression (PTGS2; log2FC = 3.63, pAdj = 0.008). Over expression of Cox-2, which synthesized many different prostanoids under inflammatory conditions, leads to decreased apoptosis by elevating anti-apoptotic factors such as Bcl-2 [57–59] and increased neoangiogenesis [60]. We identified an increase in anti-apoptotic protein Bcl-2 (Log2FC = 1.97. pAdj = 5.99E-07) in CECs suggesting a mechanism for the decreased apoptosis seen in PAH.
**3) TGF/Activin pathway:** Defects in the BMPR2 signaling pathway have also been recognized in iPAH [61,62]. We did not see a decreased expression of BMPR2 in CECs but did in LECs (log2FC = -0.70, pAdj = 0.03). We have previously shown that signaling through BMPR2 is downregulated by SMAD specific E3 ubiquitin protein ligase (SMURF) that increases the degradation of the mediator SMAD 1/5/8 [61,63–65]. We found that levels of SMURF1 and 2 are increased in CECs (SMURF1; log2FC = 0.52, pAdj = 0.07: SMURF2; log2FC = 1.57, pAdj = 0.0004) and LECs (SMURF1; log2FC = 0.59, pAdj = 0.0009: SMURF2; log2FC = 1.01, pAdj = 0.02).

Higher plasma levels of activin A are present in PAH patients [43]. We found increased expression of the activin A (INHBA) in both CECs (log2FC = 2.53, pAdj = 0.04) and LECs (log2FC = 2.10, pAdj = 0.09). Noggin (NOG), a downstream product of the activin pathway, sequesters BMPs and suppresses signaling through this pathway. We found higher levels of NOG in CECs (log2FC = 4.37, pAdj = 0.02) and LECs (log2FC = 2.43, pAdj = 0.04). Twisted gastrulation BMP signaling modulator 1 (TWSG1) is another inhibitor of BMPs that is increased in CECs (log2FC = 0.905, pAdj = 0.031) [66]. Additionally, ALK5 (TGFBR1) which is a co-receptor for activin signaling was elevated in CECs (log2FC = 1.50, pAdj = 0.007) which could enhance signaling through that pathway.

**4) Gene expression alteration in other key PAH pathways**.

Many genes have been found to play a role in the pathogenesis of PAH. We saw ectonucleoside triphosphate diphosphohydrolase 1 (ENTPD1), a regulator of the pulmonary vasculature, was reduced in both CECs (log2FC = -1.41, pAdj = 0.02) and LECs (log2FC = -1.10, pAdj = 0.02). ENTPD1 is reduced in the pulmonary artery endothelium of patients with iPAH [67].

Interleukin-6,-7, -8, and CXCL1 are potent cytokines that play an important role in PAH [33,42,68,69]. We saw increased expression of the interleukin-8 (IL-8; CXCL8) in CECs (log2FC = 3.75, pAdj = 0.006) and LECs (log2 FC = 2.40, pAdj = 0.02) and elevated IL-6 in LECs (log2 FC = 1.54, pAdj = 0.02). CXCL1 was increased in CECs (log2FC = 1.77, pAdj = 0.007) and LECs (log2FC = 1.98, pAdj = 3.59E-05). IL7R, the receptor for IL-7, which is elevated in PAH [33,69], was increased in both CECs (log2FC = 4.08, pAdj = 0.0001) and LECs (log2FC = 2.38, pAdj = 0.0005) compared to cPAECs.

Dysfunction Wnt signaling is implicated in PAH [70] as reduced WNT7 or WNT5B expression leads to abnormal angiogenesis [71]. DKK1 and DKK3 are negative regulators of the Wnt pathway and were elevated in CECs (DKK3: log2FC = 1.77, pAdj = 3.42E-05; DKK1: log2FC = 2.67, pAdj = 0.0004) and LECs (DKK3: log2FC = 0.98, pAdj = 0.0001; DKK1: log2FC = 1.04, pAdj = 0.06). NEDD9 is elevated in plasma of PAH patients, promoting endothelial fibrosis [72,73]. We found that NEDD9 levels were elevated in CECs (log2FC = 2.31, pAdj = 0.003) and LECs (log2FC = 1.54, pAdj = 5.75E-06).

## Discussion

In the present study we demonstrate that CECs have the characteristic pathway abnormalities seen in PAH and constitute a good model to test relevant disease processes in individual patients. When compared to LECs from PAH patients isolated at end-stage disease, CECs have a smaller number of DEGs with important similarities but some differences. Using our methodology, CECs were relatively easy to obtain from small amounts of blood, with the ability to culture them ~ 60% patients. This initial step for establishing an ex-vivo cell model for PAH is critical to test critical PAH pathways that can help understand the pathophysiology of the disease and move us closer to the optimal goal of personalized medicine in PAH.

Other approaches to generate endothelial cells from PAH patients involve generation of iPSCs or isolation from the balloon of pulmonary artery catheters [22–29]. Generation of iPSCs requires virus transformation of skin fibroblasts taken by biopsy. These iPSC are then transformed into endothelial cells using a mixture of different growth factors [27–29,74–77]. This process is lengthy, costly, and technically challenging and may not completely mimic the endothelial cells present in PAH patients as the process may introduce mutations and/or alter cellular transcriptomics/phenotype. There is also the possibility of lingering presence of viral fragments and the need for specialized viral handling space. However, it would be useful to make the comparison with endothelial cells generated by iPSCs, CECs and LECs to determine the usefulness in advancing PAH research.

The other alternative to isolate endothelial cells from the balloon of pulmonary artery catheter. Unfortunately, we were unsuccessful in recovering endothelial cells using this methodology, despite many modifications of our protocol (changing manufacturer of pulmonary artery catheter, longer or recurrent balloon inflation, using different media and temperature for transport, use of enzymatic product to dislodge cells from the balloon, collection with residual blood, balloon inflation while in the media, etc.). This led us to develop an alternative approach to isolate endothelial cells from PAH patients. CECs isolated by our methodology may be similar as endothelial cells isolated from the pulmonary artery balloon by other investigators, but this would require further investigation.

Although many of the important genes involved in PAH are similar in CECs and LECs, differences between them may be due to several factors, 1) number of samples analyzed with heterogeneity in disease pathways among PAH patients, 2) cells were obtained at different stages of the disease, as LECs reflect end-stage disease process that could be affected by additive mutations, epigenetics and PAH treatments, 3) cells may be different subtypes or diverse circulations [78]. Although we isolated our CEC from blood obtained from the pulmonary artery, we cannot be certain that our CECs originate in the pulmonary circulation. It is likely that PAH specific pathway abnormalities are present in a variety of endothelial cells and not just those originating from the pulmonary circulation. There is growing supports that PAH is a systemic disease [79], therefore even if our CECs are from another circulation, they clearly still manifest specific PAH pathway abnormalities, and therefore are of great value.

We have established that the CECs contain many of the defects characteristics of PAH and can be used to study the different aspects of the disease. These include pathways abnormalities involving the vascular tone, inflammation, angiogenesis, fibrosis, and thrombosis. Isolation of CECs will allow for deeper understanding of the pathogenesis of PAH and can be used as models to determine the interpatient variability. In CECs we see many of the genes that have been found to altered in PAH and are not current targets for drugs, including the Wnt pathway (DKK1, DKK3), plurinucleate pathways (ENTPD1), and the inflammatory pathways (IL6, 7, 8, CXCL1). CECs may provide a critical substrate to understand the interplay between known and novel PAH pathways.

The CECs would also greatly aid in achieving the goal of precision medicine in PAH, since current and future treatment pathways can be thoroughly assessed ex-vivo. This unique investigation can provide valuable guidance to determine the best treatment/s in every patient. Decades of research in PAH have produced many PAH-specific medications, that predominantly act at different points of the four disease pathways (nitric oxide, prostacyclin, endothelin and BMP/activin pathways). Current treatment guidelines direct PAH treatment selection based on risk stratification [2]. More than ever, we need to develop tools to base PAH treatment on mechanistic information and not only risk of complications. This patient specific treatment selection has the potential to reduce costs, minimize drug interactions and side effects, while improving the quality of life of our PAH patients [10–12,80].

All current treatment pathways, besides endothelin, were identified in our CECs and showed the alterations expected in the disease. In the NO pathway, we observed reduced levels of CAV1 and increased expression of PDE5A which reduces the NO signaling. In the prostacyclin pathway we noted a decrease in PTGIR and increase in EP4, EP1, thromboxane receptor and Cox-2; affecting the prostacyclin signaling. In the BMP/activin pathway we found factors that decrease BMP signaling, i.e. SMURF1 and 2, and noggin, as well as factors that increase activin signaling, i.e. activin A, and ALK5. As we have previously shown, there is heterogeneity in the pathways involved, which is essential to make informed treatment decisions. For example, prostacyclin agonists target the PGI_2_ but also activate several other prostaglandin receptors [67] (PTGIR, EP1, EP2, and EP4), which may impact treatment response and side effects of the prostacyclin analogue used.

Our study has limitations including a) transcriptomic analysis was performed in a small number of samples, potentially not detecting certain molecular differences between cells, and under recognizing interpatient variability, b) the origin of our CECs cannot be fully established since they were isolated from blood taken from the pulmonary catheter during RHC and there are no biological markers that would definitely attribute them to the pulmonary circulation, c) since the cellular isolation techniques are dissimilar, some of the differences observed may be due to the methodology used; however, we are focusing not only on the differences among cell lines, but predominantly on similarities regarding PH pathway abnormalities, d) culture of these CECs is only possible in ~60% of the cases, e) the endothelin pathway could not be evaluated in CECs, and f) no functional studies were performed, but are currently planned for the next stages of this research. Regardless of these limitations, this is the first study that comprehensively tested CECs isolated from a small amount of blood obtained from the pulmonary artery at the time of RHC in patients with PAH. We have shown significant differences between CECs and cPAECs in the characteristic PAH pathways. Even if the origin of our CECs is not completely clear, they carry characteristic PAH abnormalities, making them a useful cellular model to study PAH. We are currently making modifications to our protocol to increase the CECs recovery rate.

## Conclusions

In conclusion, we have described a simple method to isolate CECs from pulmonary arterial blood, providing an excellent opportunity to investigate abnormalities in PAH specific pathways, identify new pathways responsible for the pathogenesis of the disease, and guide therapeutic decisions, moving the field towards the goal of precision medicine in PAH. For the first time, this approach will allow us to determine possible temporal changes associated with the disease process, discerning events that happen at early versus late stages of PAH.

## Supporting information

S1 Table Data quality summary. Data quality summary for the raw and clean Reads for the RNA-seq.
(XLSX)

S2 Table Classification of raw reads. Classification of the raw reads for the CEC, LEC and cPAEC for RNA-seq data.
(XLSX)

S3 Table MapStat summary. Summary of the total reads one ones mapped to unique or multiple sites.
(XLSX)

S4 Table Distribution of sequencing reads of all samples to exons, introns and intergenic regions. Distribution of sequencing reads to exons, introns and intergenic regions for all the CEC, LEC and cPAEC samples undergoing RNA-seq analysis.
(XLSX)

S5 Table CEC FPKM data. FPKM data for all genes found in CEC.
(XLSX)

S6 Table LEC FPKM data. FPKM data for all genes found in LEC.
(XLSX)

S7 Table Endothelial cells FPKM data for CEC and LEC. FPKM data Specific for the endothelial cell markers.
(XLSX)

S1 File Supplemental methods. Addition information about the processing of the RNA seq data.
(DOCX)
