# Supplementary material for: Characteristic disease defects in circulating endothelial cells isolated from patients with pulmonary arterial hypertension
Source: PLoS One. 2024 Oct 28;19(10):e0312535. doi: 10.1371/journal.pone.0312535 (PMC11516004; doi:10.1371/journal.pone.0312535)
Supplement: S1 File — Addition information about the processing of the RNA seq data. (DOCX) [file pone.0312535.s008.docx]

**S1_file**

**Supplemental Methods**

**Workflow of RNA sequencing projects**

RNA sequencing via Illumina platforms, is based on the mechanism of SBS (sequencing by synthesis), and offers a wide range of benefits on high throughput and high accuracy out of low sample requirements. This method can be a powerful tool for researching RNA transcriptional activity.

**RNA sequencing projects are carried out as follows:**


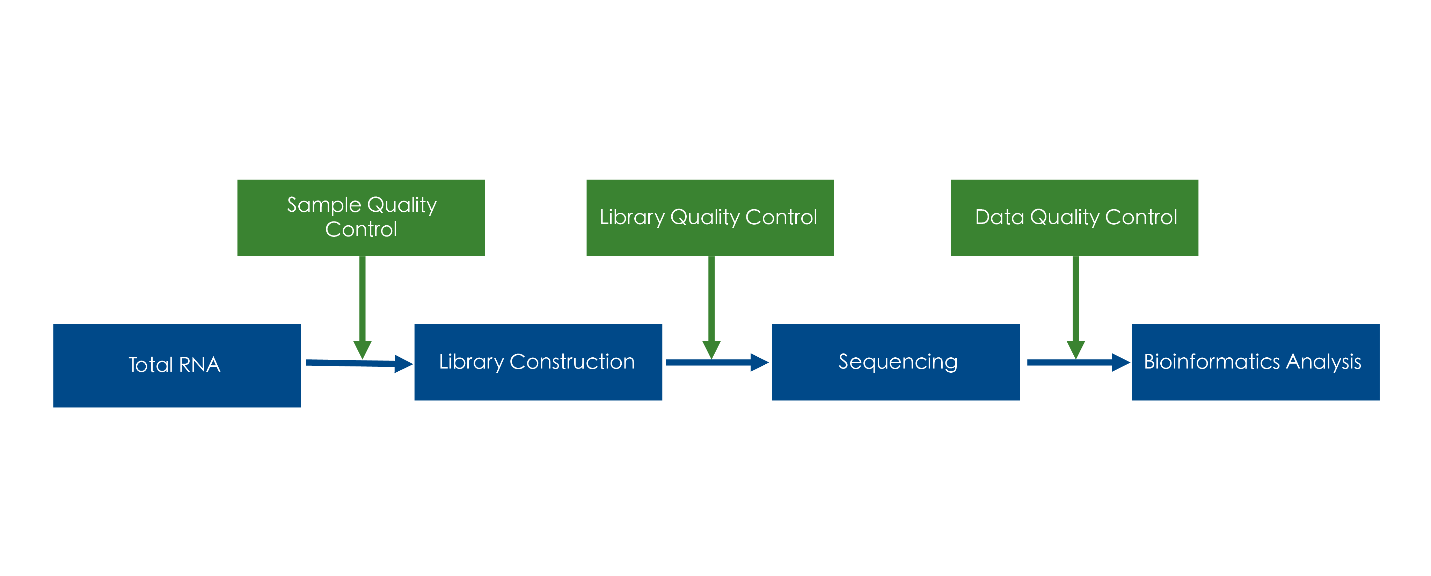


**1 Analysis Pipeline**

This is the workflow for mRNA sequencing data using standard bioinformatic analysis with a well-annotated reference genome:


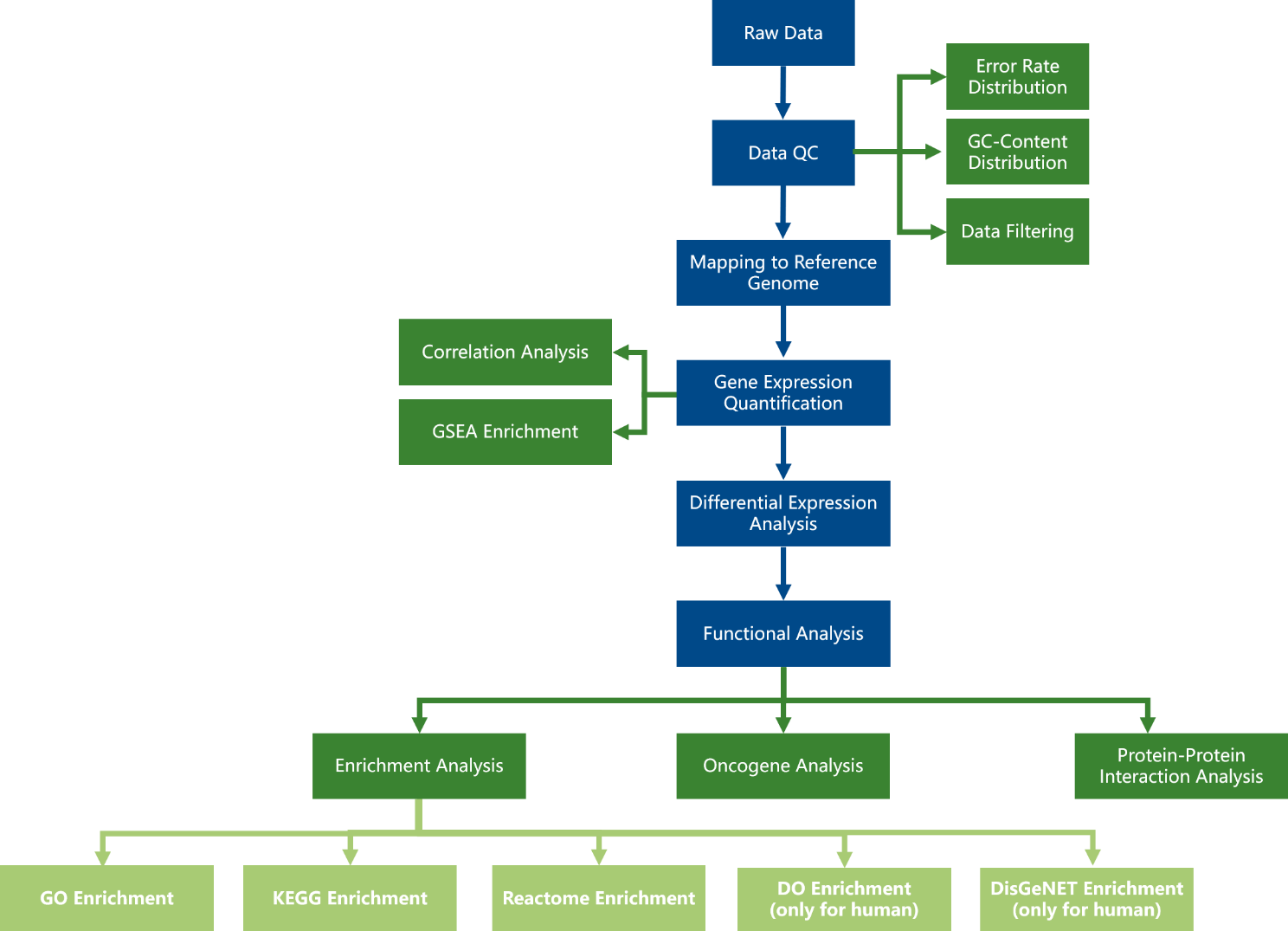


**2 Project Results**

**2.1 Raw data**

The original image data file from high-throughput sequencing platforms (using Illumina) is transformed to sequenced reads (called Raw Data or Raw Reads) by CASAVA base recognition (Base Calling). Raw data containing sequences of reads and corresponding base quality are stored in FASTQ(fq) format files.

**2.2 Data Quality Control**

**2.2.1 Error Rate**

The error rate for each base is transformed by the Phred score as in equation Qphred = -10log10(e) where "e" represents sequencing error rate, "Qphred" represents base quality values of Illumina platforms.


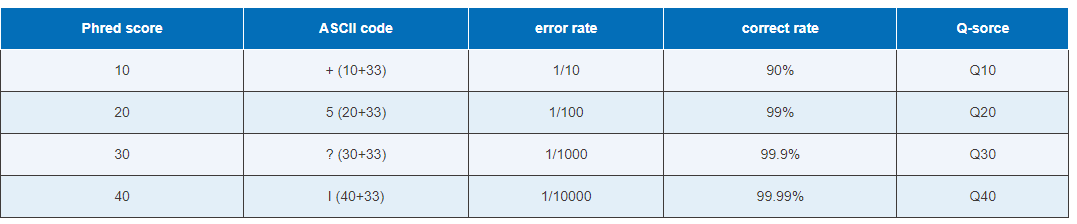


For RNA-seq technology, sequencing error rate distribution can be featured as below:

(1) Error rate increases with the sequencing reads for consumption of sequencing reagent. It is common in the Illumina high-throughput sequencing platform (Erlich Y, Mitra PP et al.2008; Jiang L, Schlesinger F et al.2011.)

(2) The first six bases have a relatively high error rate due to the incomplete binding of random hexamers used in priming cDNA synthesis (Jiang et al.). In general, a single base error rate should be lower than 1%. **See Table S1 for summary of the quality control for specific error rates for the samples used.**

**2.2.2 GC content distribution**

GC content distribution detects potential AT/GC separation, which affects subsequent gene expression quantification. In view of random fragmentation and biological law of G/C-A/T content, G and C, A and T should be respectively equal, and the content should be stable throughout the entire sequencing process for non-stranded library. A large variation of sequencing error in the first 6-7 bases is allowed considering the use of random primer in library construction, in which it is normal that the first few bases have certain preference in existing high-throughput sequencing technology**. See Table S1 for summary of the quality control for GC content for the samples used.**

**2.2.3 Data filtering**

The sequencing reads/raw reads often contain low quality reads or reads with adaptors, which will affect the quality of downstream analysis. To avoid this, it is necessary to filter the raw reads and obtain the clean reads.

**Raw reads filtering was as follows:**


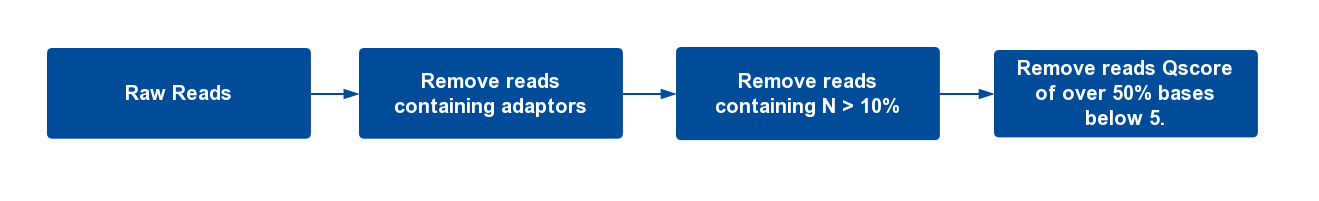


1. Remove reads with adaptor contamination.
2. Remove reads when uncertain nucleotides constitute more than 10 percent of either read (N > 10%).
3. Remove reads when low quality nucleotides (Base Quality less than 5) constitute more than 50 percent of the read.

**Table S2 contains Classification of Raw reads.**

**2.2.5 Data Quality Control**

Novogene uses a high standard of sequencing quality. Generally, the error rate of a single base should be lower than 1%. In some special cases, the maximum error rate of a single base should not be greater than 6%.

**Table S1 contains the summary of the Data quality of the samples used in the analysis.**

**2.3 Alignment**

Mapping clean reads to the reference genome or the transcriptome is the basis of the following analysis. For human with completed annotation, when differential expression gene (quantification) analysis is needed, the raw data maps reads to the transcriptome directly.

STAR software is used to accomplish the mapping. The alignment by STAR precisely and effectively performs positioning junction reads for RNA sequencing data analysis. This process is shown below:


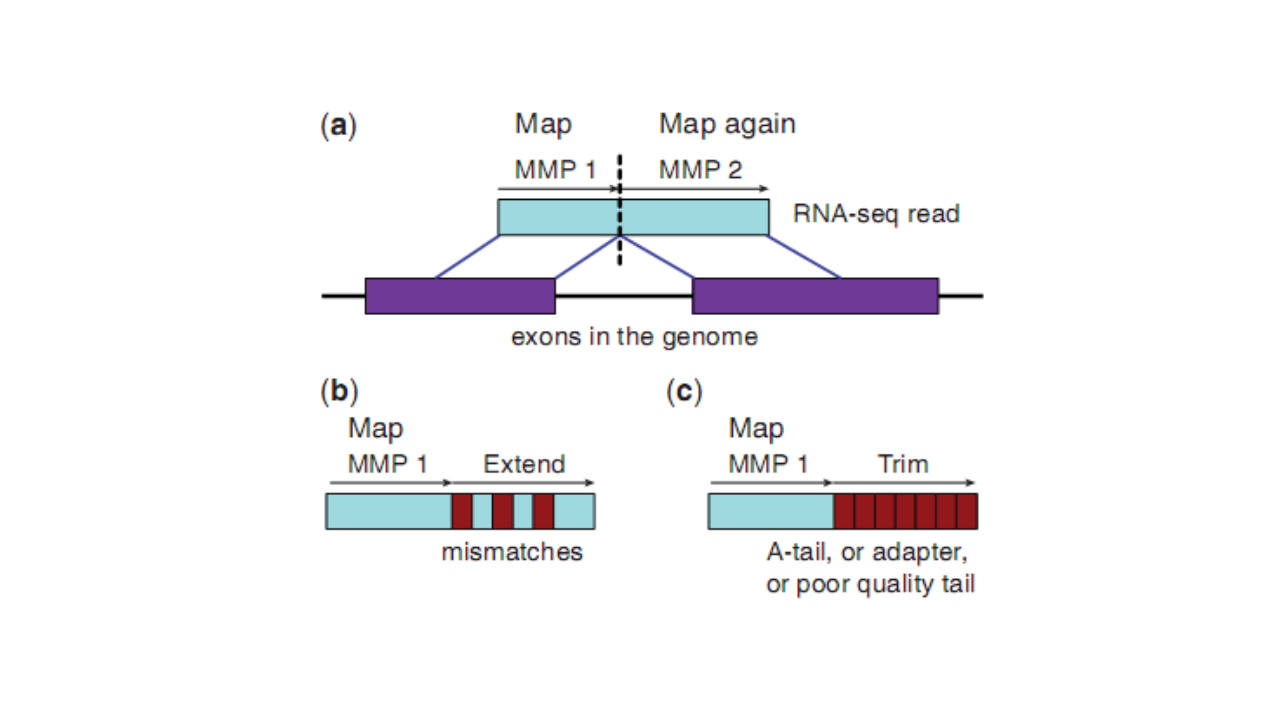


**2.3.2 Reads Distribution in Reference Genome**

Mapped regions can be classified as exons, introns, or intergenic regions. Exon-mapped reads should be the most abundant type of read when the reference genome is well-annotated. Intron-reads may be derived from pre-mRNA contamination or intron-retention from alternative splicing. Reads mapped to intergenic regions are mainly attributed to weak annotation of the reference genome.

**2.4 Gene expression level analysis**

Gene expression level analysis is the important in RNA-seq experiments. Gene expression level is calculated by the number of mapped reads.

**The data are summarized in the Table S3 (MapStat_summary) and Table S4 (Distribution of sequence reads to exons, introns and intergenic regions)**

**2.4.1 Gene Expression Quantification**

The abundance of transcript reflects gene expression level directly. In RNA-seq experiments, gene expression level is estimated by the abundance of transcripts (count of sequencing) that mapped to genome or exon. Read counts is proportional to gene expression level, gene length and sequencing depth. FPKM (Fragments Per Kilobase of transcript sequence per Millions base pairs sequenced) is the most common method of estimating gene expression levels, which takes the effects into consideration of both sequencing depth and gene length on counting of fragments (Mortazavi et al. 2008). **The data are summarized in the table S5 and S6.**

**2.5 Differential Gene Expression Analysis**

Readcount obtained from Gene Expression Analysis are used for differential expression analysis.

**2.5.1 Result of Differential Expression Analysis**

For samples with biological replicates, differential expression analysis of two conditions/groups was performed using the DESeq2 R package (Anders et al., 2010). It provides statistical routines for determining differential expression in digital gene expression data using a model based on the negative binomial distribution. Therefore, if the readcount of the i-th gene in j-th sample is Kij, there is: **Kij ～ NB(μij,σij2)**

The resulting P values were adjusted using the Benjamini and Hochberg's approach for controlling the false discovery rate (pAdj).
